# Supplementary material for: Development of Dual-Targeted Mixed Micelles Loaded with Celastrol and Evaluation on Triple-Negative Breast Cancer Therapy
Source: Pharmaceutics. 2024 Sep 6;16(9):1174. doi: 10.3390/pharmaceutics16091174 (PMC11435154; doi:10.3390/pharmaceutics16091174)
Supplement: Supplementary file 1 [file pharmaceutics-16-01174-s001.zip › pharmaceutics-3153495-supplementary.pdf]

pharmaceutics

MDPI

Supplementary Materials

The development of dual-targeted mixed micelles loaded with celastrol and evaluation on triple-negative breast cancer therapy

Siying Huang,[a] Simeng Xiao,[a] Xuehao Li,[a] Ranran Tao,[a] Zhangwei Yang,[a] Ziwei Gao[a], Junjie Hu,[a,b] Yan Meng,[a,b] Guohua Zheng,[a,b*] Xinyan Chen[a,b*]

1. Characterization of HA-Chol

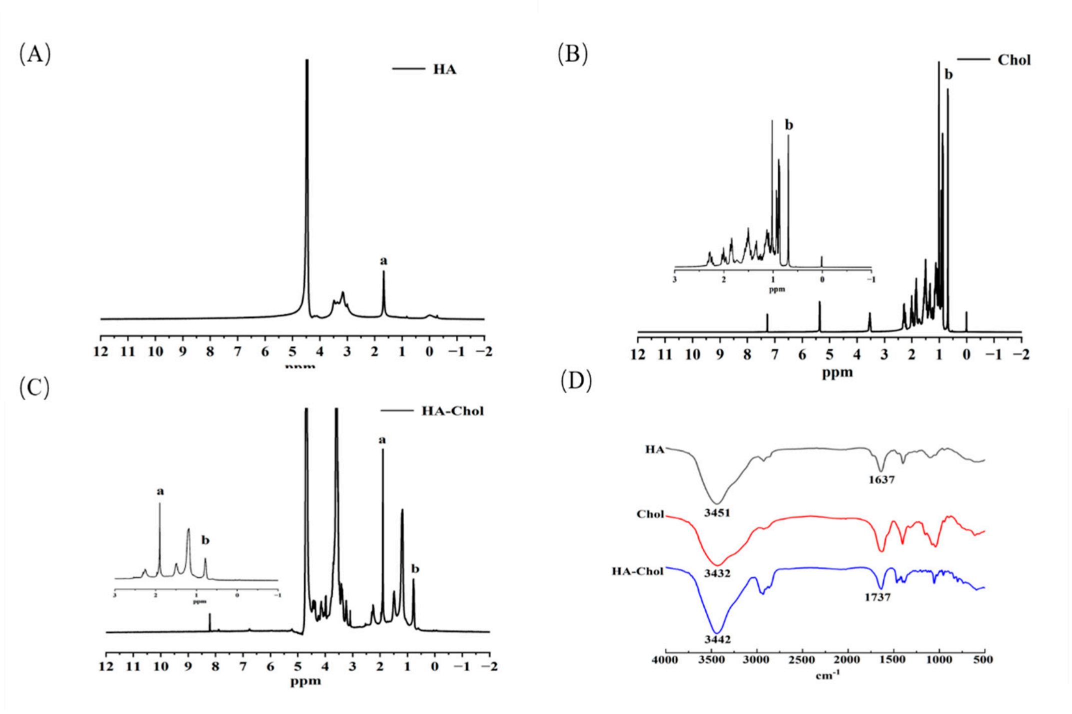

Figure S1. (A) ^{1}H NMR spectrum of HA in D_2O solution. (B) ^{1}H NMR spectrum of Chol in D_2O solution. (C) ^{1}H NMR spectrum of HA-Chol in D_2O solution. (D) FTIR spectra of HA-Chol, Chol, and HA.

The characteristic peak representing -CO-CH_3 group of HA was marked with a in Figure S1A, consistent with the literature reports. The peaks marked with b in Figure. S1B were the characteristic peaks of Chol, which were δ (ppm) 0.59,1.02 (s, 3H, CH_3), 0.87 (d, 6H, CH_3), 0.92 (d, 3H, CH_3), 1.84~2.01 (m, 5H). The peaks of HA-Chol at 0.79 ppm and 1.9 ppm were ascribed to the protons of methylene (Figure. S1C), indicating that Chol was successfully linked to HA. The graft rate of cholesterol was calculated from the relative integrated intensities of the methyl protons in cholesterol (0.79 ppm) and the methyl protons in hyaluronic acid (1.9 ppm), and the result showed that the graft rate was about 30%. The absorption peak at 2700~3000 cm^{-1} was the saturated C-H stretching vibration, which was the characteristic peak of Chol, and also appeared in the FT-IR spectrum of HA-Chol (Figure. S1D). Simultaneously, compared with the stretching vibration band of the

carboxyl group of HA at 1637 cm^{-1}, the stretching vibration band of the carbonyl group of HA-Chol obviously increased to 1737 cm^{-1}, showing that HA was bound to Chol by esterification.

2. Screening the molar ratio of TPP-Chol to HA-Chol

Table S1. Physicochemical properties of the mixed micelles prepared at different molar ratios (n=3).

TPP-Chol: HA-Chol (molar ratio)	Size in diameter/nm	Polydispersity Index	Zeta potential/mV	Encapsulation efficiency/%
1:1	133.7±3.7	0.160±0.02	-18.9±0.6	96.51±2.96
1:2	178.5±0.6	0.300±0.02	-22.8±0.4	41.13±2.96
2:1	113.9±1.2	0.472±0.03	-25.3±0.4	78.85±0.27

When the molar ratio of TPP-Chol to HA-Chol was 1:1, the encapsulation rate of celastrol was the highest, exceeding 90% (Table S1). In addition, at this molar ratio, the particle size of mixed micelles was small, and the polydispersity index was below 0.2, showing more uniform particle size dispersion. Therefore, the optimal molar ratio of TPP-Chol to HA-Chol was 1:1.

3. Screening the stirring speeds and stirring time in the preparation process of mixed micelles

Table S2. Physicochemical properties of the mixed micelles stirred for 60 minutes at different speeds (10 g, 28 g, 58 g, n=3).

Stirring speeds	Size in diameter/nm	Polydispersity Index	Zeta potential/mV	Encapsulation efficiency/%
10 g	93.0±2.0	0.184±0.02	-18.7±0.3	88.26±0.42
28 g	117.2±2.6	0.451±0.04	-30.4±0.5	60.60±0.20
58 g	287.5±2.6	0.254±0.02	-30.4±0.8	61.72±0.19

Table S3. Physicochemical properties of the mixed micelles prepared at a stirring speed of 10 g for different times（15 min, 30 min and 60 min，n=3).

Stirring time	Size in diameter/nm	Polydispersity Index	Zeta potential/mV	Encapsulation efficiency/%
15 min	164.5±3.3	0.132±0.02	-20.9±0.6	81.47±1.76
30 min	162.9±5.8	0.141±0.02	-20.9±0.6	90.31±1.48
60 min	131.4±1.7	0.156±0.01	-19.7±0.6	97.21±1.27

It can be found that the mixed micelles exhibited a smaller particle size and higher encapsulation efficiency of over 85% when the stirring speed was 300 r/min, and the stirring time was 60 min, so the optimal preparation process of the mixed micelles was TPP-Chol to HA-Chol molar ratio of 1:1, stirring speed of 300 r/min, and stirring time of 60 min.

4. The standard curve of celastrol

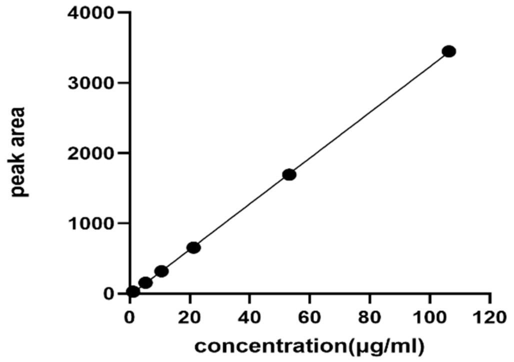

Figure S2. The standard curve of celastrol

According to Figure S2, a linear regression equation can be obtained for the peak area A and the celastrol concentration C (A=35.30C-7.366, R^2=0.9999). The results indicated that there was a good linear relationship between the celastrol concentration and peak area under the celastrol concentration range of 1.01-101.2 μg/mL, which met the requirements of the content determination method.
